# Development of nuclear SSR and chloroplast genome markers in diverse *Liriodendron chinense* germplasm based on low-coverage whole genome sequencing

**DOI:** 10.1186/s40659-020-00289-0

**Published:** 2020-05-14

**Authors:** Bin Li, Furong Lin, Ping Huang, Wenying Guo, Yongqi Zheng

**Affiliations:** 1 State Key Laboratory of Tree Genetics and Breeding, Chinese Academy of Forestry, Beijing, China; 2 Research Institute of Forestry, Chinese Academy of Forestry, Beijing, China; 3 Key Laboratory of Tree Breeding and Cultivation of State Forestry Administration, Chinese Academy of Forestry, Beijing, China

**Keywords:** *Liriodendron chinense*, SSR, Genetic diversity, Molecular markers

## Abstract

**Background:**

*Liriodendron chinense* ranges widely in subtropical China and northern Vietnam; however, it inhabits several small, isolated populations and is now an endangered species due to its limited seed production. The objective of this study was to develop a set of nuclear SSR (simple sequence repeats) and multiple chloroplast genome markers for genetic studies in *L. chinense* and their characterization in diverse germplasm.

**Results:**

We performed low-coverage whole genome sequencing of the *L. chinense* from four genotypes, assembled the chloroplast genome and identified nuclear SSR loci by searching in contigs for SSR motifs. Comparative analysis of the four chloroplast genomes of *L. chinense* revealed 45 SNPs, 17 indels, 49 polymorphic SSR loci, and five small inversions. Most chloroplast intraspecific polymorphisms were located in the interspaces of single-copy regions. In total, 6147 SSR markers were isolated from low-coverage whole genome sequences. The most common SSR motifs were dinucleotide (70.09%), followed by trinucleotide motifs (23.10%). The motif AG/TC (33.51%) was the most abundant, followed by TC/AG (25.53%). A set of 13 SSR primer combinations were tested for amplification and their ability to detect polymorphisms in a set of 109 *L. chinense* individuals, representing distinct varieties or germplasm. The number of alleles per locus ranged from 8 to 28 with an average of 21 alleles. The expected heterozygosity (*H*_e_) varied from 0.19 to 0.93 and the observed heterozygosity (*H*_o_) ranged from 0.11 to 0.79.

**Conclusions:**

The genetic resources characterized and tested in this study provide a valuable tool to detect polymorphisms in *L. chinense* for future genetic studies and breeding programs.

## Background

*Liriodendron chinense* (Hemsl.) Sarg., one of the only two living *Liriodendron* species on the earth, is Asia’s native *Liriodendron* species, known as the Chinese tulip tree [1, 2]. Depending on fossil evidence, *Liriodendron* species were reported to become extinct in Europe due to large-scale glaciation and climate aridity during glacial phases, leaving a discontinuous distribution of *L. chinense* and its American relative, *L. tulipifera* [3]. *L. chinense* grows in central and southern China and locally in northern Vietnam. These tulip trees can grow to more than 40 m in height, and their large flowers superficially resemble tulips. The trees are therefore cultivated on other continents as ornamental trees [4]. In addition, *L. chinense* wood is hard but light and difficult to deform, making it useful for construction, ship building and furniture framing. The tree’s leaves and bark are used medicinally for dispersing cold and relieving cough [5].

*Liriodendron chinense* is a cross-pollinated plant; however, parthenogenesis exists, and gynoecium can develop without insemination, causing a low germination percentage in the natural environment. Seed reproduction of this species often requires artificial pollination, whereas seeds still have poor vitality [6]. The development and utilization of *L. chinense* germplasm resources began in the 1960s when crossbreeding of Asia and American tulip trees was successfully accomplished. The hybrids maintained parental advantages, such as peculiar leaf shape and long flowering phase; moreover, they performed even better in flower color, growth rate, insect resistance, etc. Excellent characteristics are inherited through asexual reproduction techniques, such as cuttage, grafting and tissue culture in modern *L. chinense* cultivation. Various cultivars flourished from different reproducing techniques, growing areas and genetic backgrounds. The original genetic resources that breed all kinds of cultivars may be missing due to long cultivating history, multiple market circulation and careless management. Comprehensive lineage cataloguing and genetic diversity investigation are required to supervise and protect a healthy development of tulip tree resources [7].

DNA sequences may be applied in species identification, molecular phylogeny, population genetics etc. For various kinds of DNA molecular markers, microsatellite sequences are thought to be sensitive in assessing the genetic diversity and structure of plant populations. This term refers to simple sequence repeats (SSR) markers, which are codominant, highly polymorphic, reproducible, reliable, and distributed throughout the genome. In the traditional methodology, microsatellite development involves an enriched library followed by gene cloning. Tedious lab work is time-consuming and still may not be able to produce even a small number of polymorphic loci [8, 9]. Next-generation sequencing provides a good alternative, in which the genome is fully screened literally, developing thousands of SSR candidates at one time [10, 11].

The chloroplast genome is conservatively inherited uniparentally mostly via maternal inheritance [12]. In general, with a size from 120 to 160 kb, the chloroplast genome is structurally highly conserved across land plants. The chloroplast genome in angiosperms has a circular structure of two copies of large inverted repeats (IR) separated by small (SSC) and large (LSC) single-copy regions [13, 14]. Chloroplast genome markers, such as single nucleotide polymorphisms (SNPs), indels, simple sequence repeats (SSRs), and small inversion, have been used for studying genetic and genome diversity and phylogenetic and systematic evolutionary analyses [15–20]. For example, using the whole plastid genome sequence data of wild extant Ginkgo populations revealed the deepest temporal footprint dating back to approximately 390,000 year ago [21]. Diversity and phylogenetic analyses using the chloroplast genome data revealed some selection characteristics in the chloroplast genome that Asian rice had been domesticated at least twice [22]. Phyloplastomic and network analyses clarified the taxonomic position of Pepper species (*Capsicum* spp.) [23]. Moreover, the genetic information in angiosperm chloroplasts is inherited maternally, making the chloroplast markers a good indicator of maternal ancestry. Intraspecific chloroplast sequence variation is used to investigate the population structure of *L. chinense* germplasm and is applied to guide molecular breeding.

In our study, we performed low-coverage shotgun sequencing of the four genotypes of *L. chinense* using Next Generation Sequencing (NGS) technology. The study aimed to (1) assemble the chloroplast genome of *L. chinense* and identify the chloroplast genome markers, including chloroplast SSRs, indels, and SNPs; (2) develop the nuclear SSR loci by searching in contigs for SSR motifs, design candidate SSR PCR primers, and screen for fragment length polymorphisms among different *L. chinense* individuals.

## Materials and methods

### DNA extraction and high-throughput sequencing

Four genotypes of *L. chinense* were used in this study (Table 1). *L. chinense* were obtained from Monan, Songtao, Guizhou (GZST), Jiujiang, Jiangxi (JXLS), Liping, Guizhou (GZLP), and Shuining, Hunan (HNSN) of China, representing the geographical distribution of this species. Young leaves of *L. chinense* were picked for silica gel conservation. Total genomic DNA was extracted from dried leaves using the modified CTAB method [24] and further purified via the Wizard^®^ Genomic DNA Purification Kit (A1120, Promega, USA). DNA (5 ng) amount was accurately calculated on a Qubit fluorometer and digested for library construction with the TruePrep DNA Library Prep Kit V2 for Illumina (TD502, Vazyme, Nanjing, China) in accordance with the manufacturer’s instructions. A library of 350 bp was selected for sequencing on the HiSeq 4000 platform of the Novogene genome sequencing company in Tianjin, China.

**Table 1:** Summary of the complete chloroplast genome characteristics of *L. chinense*

Genotype	GZST	JXLS	GZLP	HNSN
Locality	Songtao, Guizhou, China	Jiujiang, Jiangxi, China	Liping, Guizhou, China	Shuining, Hunan China
Raw data no.	18,356,004	13,602,045	13,189,374	13,142,198
Mapped read no.	1154,826	1,099,724	1,391,064	1,603,014
Precent of chloroplast genome reads (%)	6.29%	8.08%	10.55%	12.20%
Chloroplast gemome coverage (X)	1,087	1035	1305	1506
Accession Number in Genbank	MK887905	MK887907	MK887904	MK887906
Size (bp)	159,429	159,428	159,890	159,611
LSC (bp)	87,766	87,765	88,240	87,916
SSC (bp)	18,997	18,997	19,000	19,029
IRs (bp)	26,333	26,333	26,325	26,333
GC %	39.16%	39.16%	39.15%	39.17%

### Chloroplast genome assembly and annotation

The software Trimmomatic was employed to filter low-quality reads from the raw data [25]. The remaining high-quality reads were assembled into contigs with SPAdes 3.6.1 [26]. Chloroplast genome contigs were selected from the SPAdes assembly by using BLAST search using the published *Liriodendron* chloroplast genome as a reference (GenBank accession number: KU170538). The selected contigs were second assembled with Sequencher 5.4.5 (Gene Codes, MI, USA). Ambiguous nucleotides or gaps in the chloroplast genome sequences were further confirmed by PCR amplification and Sanger sequencing with specific primers [27]. Finally, clean reads were remapped to the draft genome sequences, yielding the sequences. The chloroplast genome annotations were performed with Plann [28]. The chloroplast genome map was drawn using Genome Vx software [29].

### Chloroplast genome marker development and validation

To develop the chloroplast genome markers and to show the intraspecific variations in *L. chinense*, the four sequenced *L. chinense* chloroplast genomes were aligned using MIFFT v7 [30] and then adjusted manually using Se-Al 2.0. [31]. The markers of single nucleotide substitutions (SNPs), indels, SSRs, and inversions in the *L. chinense* chloroplast genome were identified. SNPs were calculated using MEGA 6.0 software [32]. Variable SSRs, indels and inversions were identified in the chloroplast genomes of four *L. chinense* genotypes based on the aligned sequence matrix. Using the GZST genotype genome sequence as the standard reference, the size, location, and evolutionary direction of the chloroplast genome markers were counted.

### Nuclear SSR marker development and primer design

The GZST genotype was used to develop nuclear SSR markers. We applied MISA software [33] to detect microsatellite repeats in assembled contigs. The search parameters were fixed six di-, five tri- and tetranucleotide repeats, respectively while the minimum product size was set to 100 bp. Primer pairs were designed for all candidate loci in Primer3 software [34]. Primer size was controlled between 18 and 22 bp with an optimal size of 20 bp. The minimum primer annealing temperature was set to 60 °C, and other settings were performed with default values.

### Primer testing and polymorphism detection

First, forty-eight di- or trinucleotide repeats were tested for PCR primer universality in two tulip tree samples. When synthetizing the candidate SSR primer pairs, an 18 bp tail (5′-TGTAAAACGACGGCCAGT-3′) was added to the 5′ end of the forward primer to improve efficiency and lower cost, as described in MJ Blacket, C Robin, RT Good, SF Lee and AD Miller [35]. Each 10-μL PCR mixture contained 1 × PCR buffer (with Mg^2+^), 0.25 mmol/L each dNTP, 0.25 μmol/L each primer, 1.25 U of Taq polymerase, and 20-30 ng of DNA. The PCR program was 94 °C for 4 min, followed by 35 cycles of 30 s at 94 °C, 40 s at 55 °C, and 30 s at 72 °C, with a final step of 10 min at 72 °C. The PCR products were examined via electrophoresis in a 1% agarose gel containing ethidium bromide and were visualized using an ultraviolet transilluminator. Loci amplified to be strong bands in both samples were considered for further testing in the next step.

Second, polymorphism examination of selected repeats in previous steps was performed in eight *L. chinense* from eight different populations. Each 10-μL PCR mixture contained 1 × PCR buffer (with Mg^2+^), 0.25 mmol/L each dNTP, 0.25 μmol/L each primer, 0.25 μmol/L 18 bp tail primer modified by fluorescence (FAM (blue), HEX (green), and ROX (red)) including different colors, 1.25 U of Taq polymerase, and 20-30 ng of DNA. The amplification program was the same as above. The ABI 3730xl DNA Analyzer (Applied Biosystems, Foster, CA, USA) was used to analyze the amplified PCR fragments with the GeneScan 500 LIZ size standard (Applied Biosystems).

Finally, marker primers screened out by the first two steps were validated in all 109 samples from different populations. The amplification mixture, amplification program, and analysis of PCR fragments were the same as the second step. Genotyping data were identified, and errors were corrected by GeneMapper software version 4.0 (Applied Biosystems, Thermo Fisher, USA). Genetic analyses for polymorphic loci were performed using ATetra version1.2 [36] to calculate such parameters as the number of alleles, effective number of alleles, expected heterozygosity, observed heterozygosity and Shannon’s information index.

## Result

### Chloroplast genome assembly and genome features

Using the Illumina HiSeq 4000 system, total DNA from four genotypes of *L. chinense* were sequenced to produce 13,142,198—18,356,014 paired-end raw reads (150 bp average read length) per genotype. We obtained four chloroplast genome sequences of *L. chinense* with coverage of 1035–1506 X after de nova assembly. The chloroplast genome sequences were deposited in GenBank (Table 1).

**Fig. 1:**
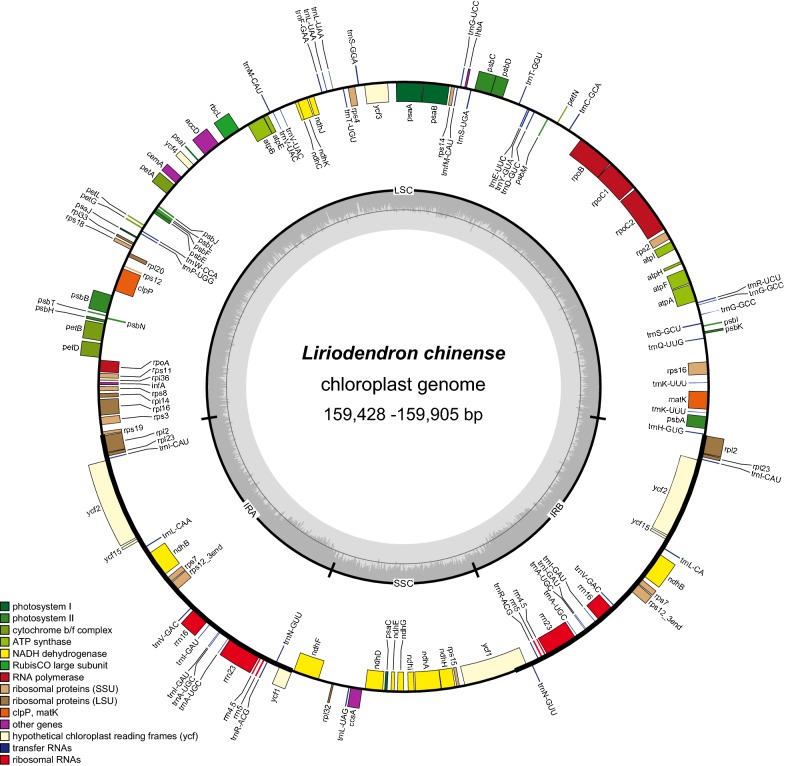
Chloroplast genome map of *L. chinense*. Genes drawn outside of the circle are transcribed clockwise, while those inside are counterclockwise. Small single copy (SSC), large single copy (LSC), and inverted repeats (IRa, IRb) are indicated

The whole chloroplast genome sequences of the four genotypes of *L. chinense* ranged from 159,428 to 159,890 bp in length (Table 1 and Fig. 1). The chloroplast genome of *L. chinense* displayed the typical circular quadripartite structure, consisting of a pair of inverted repeat (IR) regions (26,325–26,333 bp) separated by a larger single copy (LSC) region (87,765–88,240 bp), and small single copy (SSC) region (18,997–19,029 bp). The overall GC contents were 37.8% in the LSC region, 43.2% in the IR regions, 34.3% in the SSC region, and 39.2% in the entire chloroplast genome.

Gene content and arrangement were identical in four genotypes of *L. chinense* chloroplast genomes. The *L. chinense* chloroplast genome contains 113 different genes, including 79 protein-coding genes, 30 tRNA genes and 4 rRNA genes. Ten protein-coding genes (*atpF*, *ndhA*, *ndhB*, *petB*, *petD*, *rpl2*, *rpl16*, *rpoC1*, *rps12* and *rps16*) and six tRNA genes (*trnA*-*UGC*, *trnG*-*UCC*, *trnI*-*GAU*, *trnK*-*UUU*, *trnL*-*UAA*, *trnV*-*UAC*) had a single intron, while two protein-coding genes (*ycf3*, and *clpP*) contained two introns. *matK* was located within the intron of *trnK*-*UUU* in the *L. chinense* chloroplast genomes.

### Chloroplast genome marker development

There were 45 SNPs in the four genotypes of the *L. chinense* chloroplast genome, including 26 SNPs in the intergenic regions, 3 in the intronic regions, and 16 SNPs in the coding sequences (Table 2). All detected SNPs were located in the single copy region. More than two SNPs were detected in each of three positions (*trnK*-*rps16*, *psbE*-*petL*, and *ycf1*). *Ycf1*, which had the highest number of SNPs of all the positions examined, contained four SNPs. The C to G and A to T SNPs had the lowest frequencies among the six direction types of SNPs. We designed primer pairs for amplification of all the SNPs (Additional file 1: Table S1).

**Table 2:** The patterns of SNP marker in the *L. chinense* chloroplast genome

Position	Loction	Region	Type	GZST	JXLS	GZLP	HNSN
*matK*	Exon	LSC	T/C	T	T	C	C
*matK*	Exon	LSC	T/C	T	T	C	C
*matK*-*trnK*	Spacer	LSC	A/G	A	A	G	A
*trnK*-*rps16*	Spacer	LSC	T/G	T	T	G	T
*trnK*-*rps16*	Spacer	LSC	T/C	C	C	C	T
*trnK*-*rps16*	Spacer	LSC	A/C	A	A	C	C
*rps16*-*trnQ*	Spacer	LSC	T/G	T	T	G	G
*trnQ*-*psbK*	Spacer	LSC	T/G	G	G	T	G
*psbK*-*psbI*	Spacer	LSC	T/C	T	T	C	T
*trnG*	Intron	LSC	A/G	G	G	G	A
*trnR*-*atpA*	Spacer	LSC	T/C	T	T	T	C
*atpF*	Intron	LSC	A/G	G	G	G	A
*atpF*-*atpH*	Spacer	LSC	T/C	C	C	C	T
*atpH*-*atpI*	Spacer	LSC	A/C	C	C	A	A
*atpH*-*atpI*	Spacer	LSC	C/G	G	G	G	C
*rps2*-*rpoC2*	Spacer	LSC	A/T	A	A	A	T
*rpoC2*	Exon	LSC	T/G	T	T	G	T
*rpoC1*	Exon	LSC	A/G	A	A	G	G
*rpoB*-*trnC*	Spacer	LSC	T/G	G	G	T	G
*trnT*-*psbD*	Spacer	LSC	T/G	G	G	G	T
*ndhC*-*trnV*	Spacer	LSC	A/C	C	C	C	A
*trnV*	Intron	LSC	A/T	T	T	A	A
*atpB*	Exon	LSC	T/C	T	T	C	C
*accD*-*psaI*	Spacer	LSC	T/G	G	G	T	G
*psaI*-*ycf4*	Spacer	LSC	A/G	G	G	A	A
*ycf4*-*cemA*	Spacer	LSC	T/G	G	G	T	G
*psbE*-*petL*	Spacer	LSC	A/G	A	A	A	G
*psbE*-*petL*	Spacer	LSC	A/G	G	G	G	A
*psbE*-*petL*	Spacer	LSC	A/C	C	C	C	A
*rpl20*-*rps12*	Spacer	LSC	A/C	A	A	C	A
*psbB*	Exon	LSC	A/C	A	A	C	C
*rps11*-*rpl36*	Spacer	LSC	A/C	A	A	C	C
*rpl14*	Exon	LSC	A/T	A	A	T	T
*ndhF*	Exon	SSC	A/C	A	A	C	C
*ndhF*	Exon	SSC	T/C	C	C	T	C
*ndhF*-*rpl32*	Spacer	SSC	A/C	C	C	C	A
*ndhF*-*rpl32*	Spacer	SSC	A/C	A	A	C	C
*rpl32*	Exon	SSC	T/G	G	G	G	T
*psaC*-*ndhE*	Spacer	SSC	A/C	A	A	C	A
*ndhA*	Exon	SSC	A/G	G	G	A	G
*ndhH*	Exon	SSC	T/G	T	T	G	G
*ycf1*	Exon	SSC	T/C	T	T	C	C
*ycf1*	Exon	SSC	T/C	C	C	T	C
*ycf1*	Exon	SSC	A/G	A	A	G	G
*ycf1*	Exon	SSC	A/G	A	A	A	G

We identified 17 indels among the four genotypes of the *L. chinense* chloroplast genome (Table 3, Additional file 2: Table S2). All indels were found in noncoding regions. Most indels were located in the single copy regions. The size of the indels ranged from 2 to 458 bp. The largest indel (458 bp), in *ndhC*-*trnV*, was a deletion in chloroplast genome of the GZST and JXLS genotypes. The other two larger indels (> 100 bp) were found in the *ndhC*-*trnV* and *petN*-*psbM* regions.

**Table 3:** The indels makers in the *L. chinense* chloroplast genome

Position	Loction	Region	Length (bp)	GZST	JXLS	GZLP	HNSN
*matK*-*trnK*	Spacer	LSC	9	Insertion	Insertion	Deletion	Insertion
*trnK*-*rps16*	Spacer	LSC	15	Deletion	Deletion	Insertion	Insertion
*rps16*-*trnQ*	Spacer	LSC	2	Deletion	Insertion	Insertion	Insertion
*rps16*-*trnQ*	Spacer	LSC	24	Insertion	Insertion	Insertion	Deletion
*trnG*-*trnR*	Spacer	LSC	9	Insertion	Insertion	Insertion	Deletion
*petN*-*psbM*	Spacer	LSC	153	Insertion	Insertion	Insertion	Deletion
*trnE*-*trnT*	Spacer	LSC	5	Deletion	Deletion	Insertion	Deletion
*trnfM*-*rps14*	Spacer	LSC	30	Insertion	Insertion	Deletion	Deletion
*ndhC*-*trnV*	Spacer	LSC	126	Insertion	Insertion	Insertion	Deletion
*ndhC*-*trnV*	Spacer	LSC	458	Deletion	Deletion	Insertion	Insertion
*clpP*	Intron	LSC	3	Insertion	Insertion	Insertion	Deletion
*petD*-*rps11*	Spacer	LSC	15	Deletion	Deletion	Insertion	Deletion
*rpl16*	Intron	LSC	8	Deletion	Deletion	Insertion	Insertion
*trnN*-*ycf1*	Spacer	IR	9	Insertion	Insertion	Deletion	Insertion
*ccsA*-*ycf1*	Spacer	SSC	22	Deletion	Deletion	Deletion	Insertion
*ndhE*-*ndhG*	Spacer	SSC	6	Deletion	Deletion	Deletion	Insertion
*ycf1*-*trnN*	Spacer	IR	9	Insertion	Insertion	Deletion	Insertion

Forty-nine SSR loci showed polymorphism after in silico comparative analysis among the four genotypes of the *L. chinense* chloroplast genome (Additional file 1: Table S1). All polymorphic SSR loci were located in noncoding regions. Thirty-seven regions harbored SSRs; the *trnG* intron had the highest number of SSRs (three), followed by *trnH*-*psbA*, *rps16*-*trnQ*, *atpF*-*atpH*, *atpH*-*atpI*, *rps2*-*rpoC2*, *psbM*-*trnD*, *trnE*-*trnT*, *ycf4*-*cemA*, and *rpl32*-*trnL*, all of which had two SSRs. Mononucleotide motifs were the most abundant type of repeat (95.92%). Furthermore, almost all SSR loci were composed of A or T, which contributed to the bias in base composition (A/T; both 60.8%) in the chloroplast genomes of *L. chinense*. We designed primer pairs for amplification of all the SSRs (Additional file 3: Table S3).

Moreover, a total of five small inversions were uncovered based on the sequence alignment of the four chloroplast genomes (Table 4, Additional file 4: Table S4). Of which these inversions, three were located in the LSC region and two were in the SSC region. All inversions were accompanied by a pair of inverted repeats immediately flanking the inversion. The inversions were from 3 to 23 bp and the franking repeats were from 17 to 29 bp in length. The two small inversions from *petA*-*psbJ* and the inversion from *trnH*-*psbA* only occurred in the GZLP genotype. The small inversions in *rpl32*-*trnL*, and *ccsA*-*ycf1* occurred in the GZLP and HNSN genotypes.

**Table 4:** The locations, directions, and lengths of small inversions

Location	Region	Length of inversions (bp)		Direction of the small inversions			
		Length of inversion	Length of inverted repeat	GZST	JXLS	GZLP	HNSN
*trnH*-*psbA*	LSC	8	21	No	No	Yes	No
*petA*-*psbJ*	LSC	10	17	No	No	Yes	No
*petA*-*psbJ*	LSC	12	29	No	No	Yes	No
*rpl32*-*trnL*	SSC	3	22	No	No	Yes	Yes
*ccsA*-*ycf1*	SSC	23	17	No	No	Yes	Yes

### Nuclear microsatellite marker development

The paired end reads of the GZST genotype were qualitatively assessed and assembled with SPAdes 3.6.1. There were 161,179 contigs assembled and the contig length ranged from 150 bp to 113,929 bp.

**Fig. 2:**
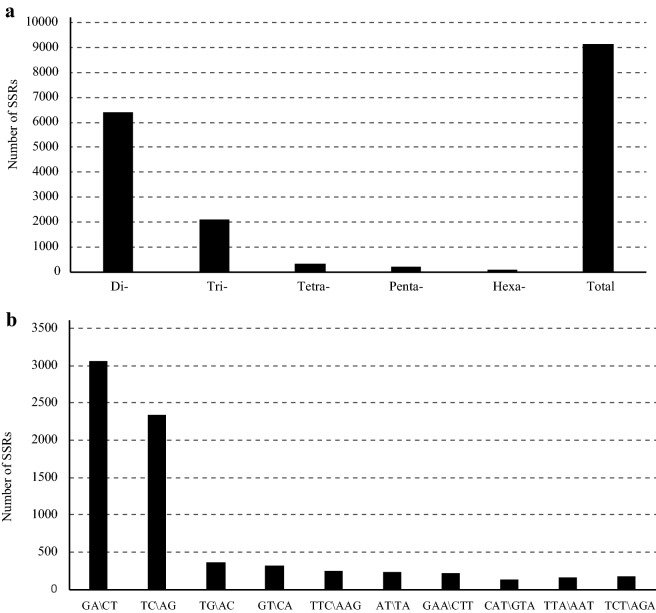
Distribution and type of SSRs in *L. chinense*. **a** Number of different SSRs types. **b** Number of identified SSR motifs in different repeat class types

A total of 9155 SSRs were discovered using MISA from the assembled contigs. These SSRs included 6417 di-, 2115 tri-, 312 tetra-, 219 penta- and 92 hexanucleotide repeats, which corresponded to 70.09%, 23.10%, 3.41%, 2.39%, and 1.00% of total SSRs, respectively (Fig. 2a). According to the distribution of microsatellites, SSR frequency and density varied with motif length, as motif length increased (from mono- to hexanucleotide repeats). Dinucleotide repeats are more common than the higher order motif, which is in agreement with previous research examining other wood plants. The ten most frequent motif types in the *L. chinense* genome (Fig. 2b) were five dinucleotides (GA/CT, TC/AG, TG/AC, AT/TA, GT/CA, AT/TA), and five trinucleotides (TTC/AAG, GAA/CTT, CAT/GTA, TTA/AAT, TCT/AGA).

### Primer design and evaluation

Primer3 was used to generate primer pairs targeting these SSR regions (Additional file 5: Table S5). In total, there were 5339 SSR-designed primers. We randomly selected 48 SSRs for initial validation in eight individuals. After screening in 2% agarose electrophoresis, 18 of the 48 primer pairs produced clear, unique amplification products of the expected size. Of 18 SSRs, 13 loci had polymorphic amplifications, and 5 loci were monomorphic. These polymorphic loci serve as candidate markers in the following analysis.

### Genetic diversity and relationships among genotypes

To evaluate the genetic diversity of *L. chinense*, 109 individuals were collected and analyzed using 13 primer pairs selected. Based on 13 polymorphic primer pairs, the number of alleles (*A*) per locus ranged from 8 to 28, with an average of 21. The expected heterozygosity (*H*_e_) varied from 0.1919 to 0.9344 and the observed heterozygosity (*H*_o_) ranged from 0.1101 to 0.7944 (Table 5).

**Table 5:** Characterization of the 13 polymorphic nuclear SSR markers

Loci	Forward primer sequences(5′ to 3′)	Reverse primer sequences(5′ to 3′)	Predicted size	Motif	Repeats	*A*	*H*_o_	*H*_e_	*H’*
Loci01	CGATAGCGAGAAGAGATACGGG	AGAGAAAAATCAGGCCAGTCCA	248	CA	7	16	0.3564	0.8693	2.2622
Loci02	CGGGGTCTTGATTTTGGAGAGA	CTGTAGACGTGCTCTTCCGATT	269	AG	10	8	0.3084	0.4916	1.0601
Loci03	GTTTTCTCCAATGCTCCACACC	CTCTATAGTCCTCGTGTCGCAC	253	AG	13	24	0.2909	0.9344	2.9145
Loci04	CGTTTCAAATAGGTGGGAGGGA	TGCTGTCCCAAAGCTTCACTAA	256	CT	7	21	0.7532	0.913	2.6479
Loci05	AGAGGAAGTGGAGGAAGAAGGA	TGCCCTCATTTATCTCTCTCGC	168	AG	13	12	0.1101	0.1919	0.5557
Loci06	TCGAGTTGGCGAGTAATTGTCA	TCTTTGTCGCTTTCTCTCCCTC	250	AG	14	21	0.573	0.9156	2.6439
Loci07	ATGTCCAGTCGTAGAAGGGAGA	ATCTTACAAATTCCCCCTGGGC	280	TC	13	27	0.7733	0.9023	2.7063
Loci08	CCAAGACGAGAACGATCGATCT	AAGTGAGAAAATGCACGTGGTG	157	TC	7	19	0.7113	0.8737	2.4331
Loci09	AGGGGATTACTGACGTCGAGTA	GAGTATCATAGGCCCATTACCCT	260	AG	13	28	0.3486	0.9198	2.8313
Loci10	GGATTTAGTTCGGGGAAGACGT	TAGGGCCGTTTGCAACATTTTT	236	CT	7	22	0.1972	0.9059	2.606
Loci11	CATGCCAGGCCTGTTAAAAGTC	GCTAGCTCTGACAGGCTTCTAG	179	GT	7	25	0.7944	0.9034	2.5901
Loci12	GGCACAGATCAAAAATCGCACT	CTTCCATGCCTCTCCGCCATTA	140	GA	18	24	0.7156	0.7804	1.9415
Loci13	CAACCTTCTCTGTCACCTCCG	ATAAGTAGTGGAGAGCATGCGG	145	TC	14	26	0.486	0.9221	2.8333
Mean						21	0.4937	0.8095	2.3097

*A* number of alleles, *H*_o_ observed heterozygosity, *H*_e_ expected heterozygosity, *H*’ Shannon‐Wiener diversity

## Discussion

### Nuclear SSR markers developed by NGS

Recent developments in sequencing technologies and bioinformatic analysis have provided an unprecedented opportunity to discover SSR markers of high quality and effective cost/time in nonmodel organisms about which genomic information was lacking. Moreover, this approach is also rapid and more cost-effective than traditional SSR development methods and Sanger sequencing [37, 38]. In this study, we randomly obtained partition nuclear genome sequences, and this approach was sufficient for the development of 9155 SSR markers for *L. chinense.* The density of SSRs was 1 per 7.04 kb in the *L. chinense* chloroplast genome, while in the most plant genomes, every 6.8 kb has one SSR. This density was less than the density in coffee (1/2.16 kb), and *Amorphophallus* (1/3.63 kb) [39, 40], but it was higher than that of *Arabidopsis* (1/14 kb) [41]. The variable frequency of genic and genomic SSRs may reflect a difference in their distribution in coding sequences compared to the entire genome. In the *L. tulipifera* EST data, the average frequency of SSR was 1 per 8.5 kb, which was less frequent than the genomic data [42].

Among the selected genomic SSRs, dinucleotide repeats were the most abundant (70.09%), followed by trinucleotide motifs (23.10%). One of the 10 most abundant motif types in jujube was tetranucleotide motifs (ATTT/AAAT) which was not found in *L. tulipifera* [43]. The motif length frequency differences between genomic and genic SSRs are most likely due to selection pressure on genic SSRs which reduces the fixation of mutations leading to frameshifts. Among dinucleotide repeats, AG/CT was most frequently observed (33.51%), followed by AG/TC (25.53%) which is in agreement with Xu et al. [42], who reported that AG/CT was the most frequent genic dinucleotide (57.4%) in *L. chinense*.

Several studies used expressed sequence tags (ESTs) developing SSR markers [1, 6, 42]. Compared with this study, those SSRs had the lower variable, for example, the average effective number of alleles was 3.95 to 5.93 [38, 44]. Using the low- coverage whole genome sequencing method, we quickly obtained a number of nuclear SSRs with low costs.

### Application of nuclear SSR markers

Nuclear microsatellite, with a mutation rate ranging from 10^−6^ to 10^−2^ [45], are highly polymorphic in comparison with other marker systems, which have been widely used in many living organisms including plant, insects, birds, humans and animals for different kinds of basic genetics research. There were many applications of nuclear SSR markers in plants, such as, genetic diversity and phylogenetic relationships, population and evolutionary studies, cultivar identification and marker-assisted selection, genome mapping [46].

SSR markers often are powerful system for revealing interspecific and/or intraspecific phylogenetic relationships. Several applications show nuclear SSRs have led to a better understanding of close relationships between species. Relationships among eight *Actinidia* species were resolved with SSRs [47]. The fragment length polymorphism of SSRs among the *Cucumis* accessions made it possible to distinguish three main groups [48]. The genetic diversity for germplasm collections have been assessed by SSR markers, such as apple [49], eggplant [50], walnut [51]. Belgj et al. examined the pattern of genetic variability and genetic relationships of wild olive populations in the north-western Mediterranean and indicated a degree of admixture in all the populations [52]. Evaluation of genetic diversity, genome mapping and phylogenetic relationships has resulted in information of the history process and will provide important information for breeding programs.

### Chloroplast genome variation in *L. chinense*

The chloroplast genomes of plants are a valuable resource for developing molecular markers to study intraspecies and interspecies ecolution [15, 53]. Chloroplast genomic sequences are highly conserved within species; however, nucleotide substitutions, SSRs, indels, and other microstructure mutations within these sequences can be used to elucidate the genetic diversity and guide molecular breeding [54, 55]. However, few studies have used whole chloroplast genome data to examine intraspecific diversity. In this study, we assembled chloroplast genome sequences of four accession samples from wild *L. chinense* germplasm using low-coverage NGS data.

Comparative analysis of the four chloroplast genomes of *L. chinense* revealed 45 SNPs, 17 indels, 49 polymorphic SSR loci, and five small inversions. The abundant genetic diversity could be applied to phylogenetic analysis and development of molecular markers to verify the genetic diversity of *L. chinense*. Chloroplast genome sequence diversity in *L. chinense* is relatively high compared to that reported for other plant species including *Scutellaria baicalensis* (25 SNPs, 19 indels, two individuals) [56], *Brachypodium distachyon* (298 SNPs, 53 individuals) [20], *Jacobaea vulgaris* (32 SNPs, 17 individuals) [57] and *Dioscorea polystachya* (141 SNPs, 43 indels, 24 polymorphic SSRs, six individuals) [54].

Previously, *trnH*-*psbA*, *trnL*-*F*, *rbcL*, and *matK* genes were used in evolutionary studies, and which were reported to be variation hotspots [58, 59]. Of these genes, *matK* and *rbcL* were the core DNA barcodes in plants [60]. More studies have been shown that the mutation hotspot regions in the chloroplast genome are concentrated in noncoding regions. For example, Dong et al. identified 23 highly variable chloroplast markers (4 coding regions, 2 introns, and 17 intergenic spacers) that were used to resolve phylogenies and for DNA barcoding of closely related flowering plant species [61]. Regions of particularly high variability in *L. chinense* included the LSC intergenic spacer regions *trnK*-*rps16* (five polymorphisms) and *rps16*-*trnQ* (five polymorphisms) followed by *atpH*-*atpI*, *petA*-*psbJ*, *trnG* intron and *ycf1*. *trnK*-*rps16* and *rps16*-*trnQ* have been identified as variable and underutilized regions of the angiosperm chloroplast genome suitable for intraspecific phylogenetic studies [61, 62]. *Ycf1* is the second longest gene and the most rapidly evolving chloroplast gene [63], the function of which is essential for plant viability and encodes Tic214, a vital component of the *Arabidopsis* TIC complex [64]. There were two highly variable regions (ycf1a and ycf1b) in the SSC of the *ycf1* gene [61, 63].

The polymorphisms found in this study can be used to elucidate evolutionary history such as promoting practical applications for breeding new cultivars of *L. chinense.* Furthermore, chloroplast polymorphism markers will be useful in testing maternal inheritance of the chloroplast genome, in identifying genotype differentiation and even in developing breeding programs.

## Conclusion

In this study, we obtained four chloroplast genomes of *L. chinense* from four genotypes, and identified SNPs indels, SSRs and small inversions in *L. chinense* by comparative analyses of chloroplast genomes. We also developed nuclear SSRs by low-coverage whole genome sequencing. These newly developed chloroplast genome resources and SSR markers will become useful tools for molecular genetics, genotype identification, genetic mapping, and molecular breeding of Chinese tulip tree.

## Supplementary information

**Additional file 1: Table S1.** Primers of SNP markers in the *L. chinense* chloroplast genome.

**Additional file 2: Table S2.** Primers of Indels markers in the *L. chinense* chloroplast genome.

**Additional file 3: Table S3.** SSRs identified from in silico comparative analysis of the chloroplast genomes of four *L. chinense* genotypes.

**Additional file 4: Table S4.** Primers of small inversions.

**Additional file 5: Table S5.** Nuclear SSRs identified in this study.

## Data Availability

The newly sequenced plastomes have been submitted to GenBank with Accession Numbers MK887904–MK887907.

## Abbreviation

SSR: Simple sequence repeat
SNP: Single nucleotide polymorphisms
LSC: Large single copy
SSC: Small single copy
IR: Inverted repeat
